# Characterization of *Proteus mirabilis* and associated plasmids isolated from anaerobic dairy cattle manure digesters

**DOI:** 10.1371/journal.pone.0289703

**Published:** 2023-08-10

**Authors:** Gabhan Chalmers, Rebecca E. V. Anderson, Roger Murray, Edward Topp, Patrick Boerlin

**Affiliations:** 1 Department of Pathobiology, Ontario Veterinary College, University of Guelph, Guelph, Ontario, Canada; 2 London Research and Development Centre, Agriculture and Agri-Food Canada, London, Ontario, Canada; North Carolina State University, UNITED STATES

## Abstract

*Proteus mirabilis* is an opportunistic pathogen associated with a variety of human infections, including urinary tract infections. The prevalence of *P*. *mirabilis* in foods of animal origin and in the manure by-products created in animal production is not well documented. Further, the prevalence and persistence of extended-spectrum cephalosporin (ESC) resistant *P*. *mirabilis* is largely unknown. In this study, we characterized ESC-resistant *P*. *mirabilis* recovered from various stages of dairy manure anaerobic digestion. Isolates were screened by PCR for *bla*_CTX-M_, *bla*_CMY_ and *bla*_SHV_, and antimicrobial susceptibility testing was performed. Fifty-six *P*. *mirabilis* carrying CTX-M were sequenced with short and long read sequencing technologies, and the assembled chromosomes and plasmids were compared. ESC-resistant *Proteus* was found in four of the six manure digesters, an indication that not all digesters were colonized with resistant strains. Both CTX-M-1 and CTX-M-15 plasmids were found in *P*. *mirabilis* isolates. Transfer of plasmid DNA by conjugation was also explored, with ESC-resistance plasmids able to transfer to *Escherichia coli* at high frequency. We concluded that *P*. *mirabilis* can harbour and transfer ESC-resistance genes and plasmids, and may be an overlooked reservoir of antimicrobial resistance.

## Introduction

Third and fourth generation cephalosporins, or extended-spectrum cephalosporins (ESCs) have been defined by the World Health Organization (WHO) as critically important antimicrobial agents [1]. The primary mechanisms of ESC resistance in Enterobacterales involve the production of extended spectrum *β*-lactamases (ESBLs) and AmpC β-lactamases [2]. Historically, the CMY-2 AmpC *β*-lactamase has been the main determinant of ESC-resistance found in Enterobacterales from animals in Canada; the CTX-M-type ESBLs only appeared more recently, and have now spread amongst bacteria from chicken, cattle, and swine [3–5].

*Proteus mirabilis* is a Gram-negative, facultative anaerobic rod, which can be found in soil and water. It is also part of the normal human and animal intestinal flora, along with *Escherichia coli*. It can cause disease in humans in the form of urinary tract infections and other opportunistic infections [6,7], and approximately 90% of *Proteus* infections are caused by *P*. *mirabilis* [8,9]. Almost nothing is known about it in farm animals in Canada, particularly with respect to antimicrobial resistance (AMR) and its role in the global epidemiology of ESC-resistance in Enterobacterales.

Our objectives were to study the distribution of ESC-resistant *Proteus* and to characterize them as part of a larger study involving dairy farm manure in various states of anaerobic digestion [10]. A particular emphasis was put on *bla*_CTX-M_-positive isolates and their resistance plasmids in order to provide a basis for comparisons with plasmids from other more widely studied Enterobacterales.

## Material and methods

### Bacteria isolation, antimicrobial susceptibility testing, and PCR

The study design and sampling strategy has been previously described in detail by Tran and collaborators [10] and Anderson *et al*. [11]. Briefly, anaerobic dairy cattle manure digester samples were collected at regular intervals from six farms (labelled farm 1, 2, 3, 4, 5 and 7) between November 2018 and October 2019 from the Canadian province of Ontario, resulting in 164 samples tested. Authorization for sampling was provided directly by the farmers. Farms 1 through 4 utilized two mesophilic anaerobic digesters each. Farm 5 used an additional third thermophilic anaerobic digester and farm 7 used one mesophilic digester and a thermophilic composter [10]. Manure samples were enriched in EC broth (Becton, Dickinson and Company, Sparks, MD) containing 2 mg/L of cefotaxime (Sigma-Aldrich, St. Louis, MO) overnight at 37°C [11]. A 10 μL loopful was then streaked onto MacConkey agar (Becton, Dickinson and Company) plates containing 1 mg/L ceftriaxone (Sigma-Aldrich), and non-lactose fermenting colonies were purified and identified to the species level using matrix assisted laser desorption ionization-time of flight mass spectrometry (MALDI-TOF MS; Bruker Daltonik GmbH, Bremen, Germany) before further characterization.

All *P*. *mirabilis* isolates were screened for *bla*_CMY_, *bla*_CTX-M_, and *bla*_SHV_ genes using single and multiplex PCR as previously described [12,13]. Lysates were prepared using centrifuged boiled bacterial suspensions and the supernatants used as PCR template DNA. Positive isolates were then tested for susceptibility to chloramphenicol, ciprofloxacin, compound sulfonamides, sulfamethoxazole-trimethoprim, kanamycin, gentamicin, streptomycin, tetracycline, ampicillin, ampicillin plus clavulanic acid, cefotaxime, and cefoxitin (Becton, Dickinson and Company), using the disk diffusion method following the Clinical Laboratory Standards Institute’s performance standards [14]. Only isolates with inhibition zone diameters below the resistance breakpoint were considered resistant (i.e. isolates with intermediate susceptibility were pooled with fully susceptible isolates).

### Genome sequencing

All *bla*_CTX-M_-positive isolates (*n* = 56) were sequenced using the Illumina MiSeq platform (PE150; Illumina, San Diego, CA, USA) at Genome Québec at McGill University, Montreal, Québec. Genomic DNA was prepared using the EpiCentre MasterPure DNA Purification kit (EpiCentre, Madison, WI, USA) following the manufacturer’s instructions. Long read sequencing of isolates was performed using a MinION Mk1B device (Oxford Nanopore Technologies, Oxford, United Kingdom). Sequencing libraries and barcoding preparation was performed using the SQK-LSK109 and EXP-NBD104/114 ligation and native barcoding kits (Oxford Nanopore Technologies) according to the manufacturer’s instructions. Between 8 and 16 samples were run on each of four flow cells (version FLO-MIN111), which were run until exhaustion. Basecalling of fast5 files and demultiplexing was performed using Guppy Basecaller v5.0.11 (Oxford Nanopore Technologies) with barcode trimming enabled. Hybrid assembly of short and long reads was performed using Unicycler v0.4.8 [15] and visualized with Bandage v0.8.1 [16]. Antimicrobial resistance genes and plasmid replicon types were annotated using ABRicate v0.9.8 [17] with the Resfinder and Plasmidfinder databases enabled. MOB-suite v3.0.3 (MOB_typer) was also used to determine relaxase types for each plasmid [18].

### Chromosome and plasmid analysis

A single nucleotide polymorphism (SNP) analysis was performed for all 56 chromosome assemblies using Snippy v4.4.5 [19], with *P*. *mirabilis* strain HI4320 (GenBank accession NC_010554.1) used as a reference genome. The snippy-core function was used to generate a core genome SNP alignment, and the output file (clean.full.aln) was analyzed with Gubbins v1.4.5 [20], to produce another output file (clean.core.aln). SNP-sites [21] was then used to extract the SNPs from the alignment file, which was then used to infer a maximum-likelihood phylogenetic tree using FastTree v2.2.11 [22], and was visualized with Geneious v9.1.8 (Biomatters, Auckland, New Zealand).

Circularized plasmid assemblies were verified using long reads mapped to the reference sequence, and coverage was checked visually. Plasmids sequences carrying *bla*_CTX-M_ (*n* = 55) were annotated using Prokka v1.14.6 [23]; two of these plasmids were not suitable for pairwise SNP comparison, as the IncN plasmid of isolate 259-2e and the 101kb plasmid from isolate 341-1d were too dissimilar to include, based on an initial analysis. Roary v3.13.0 [24] was used to generate a multi-FASTA alignment of the core genes common to the remaining 53 plasmid sequences, using the -e and—mafft parameters. The resulting core_gene_alignment.aln file was then used to generate a phenetic tree using FastTree, as well as SNP-sites to generate a SNP alignment file.

### Conjugation and stability

The transfer of *bla*_CTX-M-1_ and *bla*_CTX-M-15_ plasmids from *P*. *mirabilis* to *Escherichia coli* was quantified by conjugation experiments carried out in triplicate in liquid broth and on solid media at both 30°C and 37°C [25]. Isolate 240-2c (*bla*_CTX-M-15_) and isolate 259-2e (*bla*_CTX-M-1_) were used as donor strains, while the rifampicin and kanamycin-resistant *E*. *coli* strain C439 gfpCV60 [10] was used as a recipient. For conjugation in broth, 5 mL of LB broth cultures containing appropriate selective antimicrobials (2 mg/L cefotaxime or 50 mg/L kanamycin) were incubated overnight with shaking. Bacteria were then harvested by centrifugation, washed with LB broth to remove antimicrobial residues, and resuspended in 1 mL of LB broth. A 200 μL aliquot of both donor and recipient strain were added to 1.6 mL LB broth and incubated for 16 h without shaking. For solid media conjugation, bacteria from 1 mL of overnight donor culture and 100 μL of recipient culture were mixed, centrifuged, and resuspended in 100 μL of LB, then inoculated onto an approximately 2.5 cm^2^ filter paper placed on an LB plate, let to dry and incubated for 16 h. After incubation, bacteria were resuspended in 1 mL LB broth, and serial dilutions of transconjugants were selected on LB agar containing 50 mg/L rifampicin, 50 mg/L kanamycin, and 2 mg/L cefotaxime. Conjugation rates were calculated using the transconjugant to recipient ratio. Successful transfer of the *bla*_CTX-M_ plasmids was confirmed by PCR on three transconjugant colonies per plasmid type. The short-term stability of plasmids in the *E*. *coli* transconjugants was measured by repeated (6 rounds of 100 μL of culture into 9.9 mL of plain LB broth) subcultures of transconjugant isolates in LB broth. Cultures were then grown on plain LB agar, and 10 isolated colonies were tested for plasmid loss by inoculation onto media containing 1 mg/L ceftriaxone and observing growth after 16 hours.

## Results

### *Proteus mirabilis* identification and distribution of resistance

Seventy-two ESC-resistant *P*. *mirabilis* were isolated from four of the six farms (Table 1, and in more detail in S1 and S2 Tables); no resistant isolates were recovered from farm 2 and farm 5. The 72 isolates were obtained from 22 of the 164 samples tested. The majority of isolates carried *bla*_CTX-M_ (78%, 56/72), while the remaining 22% (16/72) carried *bla*_CMY-2_. All 72 isolates were resistant to tetracycline, ampicillin, and cefotaxime, and all were susceptible to ciprofloxacin. The complete susceptibility testing results and associated inhibition zone diameters are shown in S1 and S2 Tables.

**Table 1:** ESC-resistant *P*. *mirabilis* isolates characterized in this study with farm source and associated ESC resistance genes.

	Samples with ESC^R^-*P*. *mirabilis*	Isolates carrying CTX-M	CTX-M variants	CTX-M-carrying plasmid types	Isolates carrying CMY
Farm 1	4/26	3	CTX-M-15	Inc-negative	10
Farm 3	5/31	1	CTX-M-65 (chromosomal)	N/A	6
Farm 4	7/16	15	CTX-M-15	Inc-negative	0
Farm 7	16/32	37	CTX-M-15 (36), CTX-M-1 (1)	Inc-negative (36), IncN (1)	0

No ESC^R^ isolates were obtained from farm 2 samples (n = 12) or farm 5 (n = 39).

### Genome and plasmid characterization of CTX-M-positive isolates

All *bla*_CTX-M_-carrying isolates were successfully sequenced using both Illumina and Oxford Nanopore technologies, and all assemblies have been deposited in GenBank under BioProject PRJNA833106 (BioSamples SAMN26871933-SAMN26871988). The plasmid sizes, replicon types, and collocated resistance genes are listed in S1 Table. The most frequent CTX-M variant found was *bla*_CTX-M-15_ (96%), with *bla*_CTX-M-1_ and *bla*_CTX-M-65_ found in only one isolate each. The *bla*_CTX-M_ genes were generally plasmid-borne; only one isolate carried it on its chromosome (*bla*_CTX-M-65_). All *bla*_CTX-M_-positive isolates also carried the *aac(6’)-Ib-cr* gene, except for the *bla*_CTX-M-1_-positive isolate. As expected [26], this was associated with reduced susceptibility to ciprofloxacin, although not with full resistance to this critically important antimicrobial (S1 Table).

**Fig 1:**
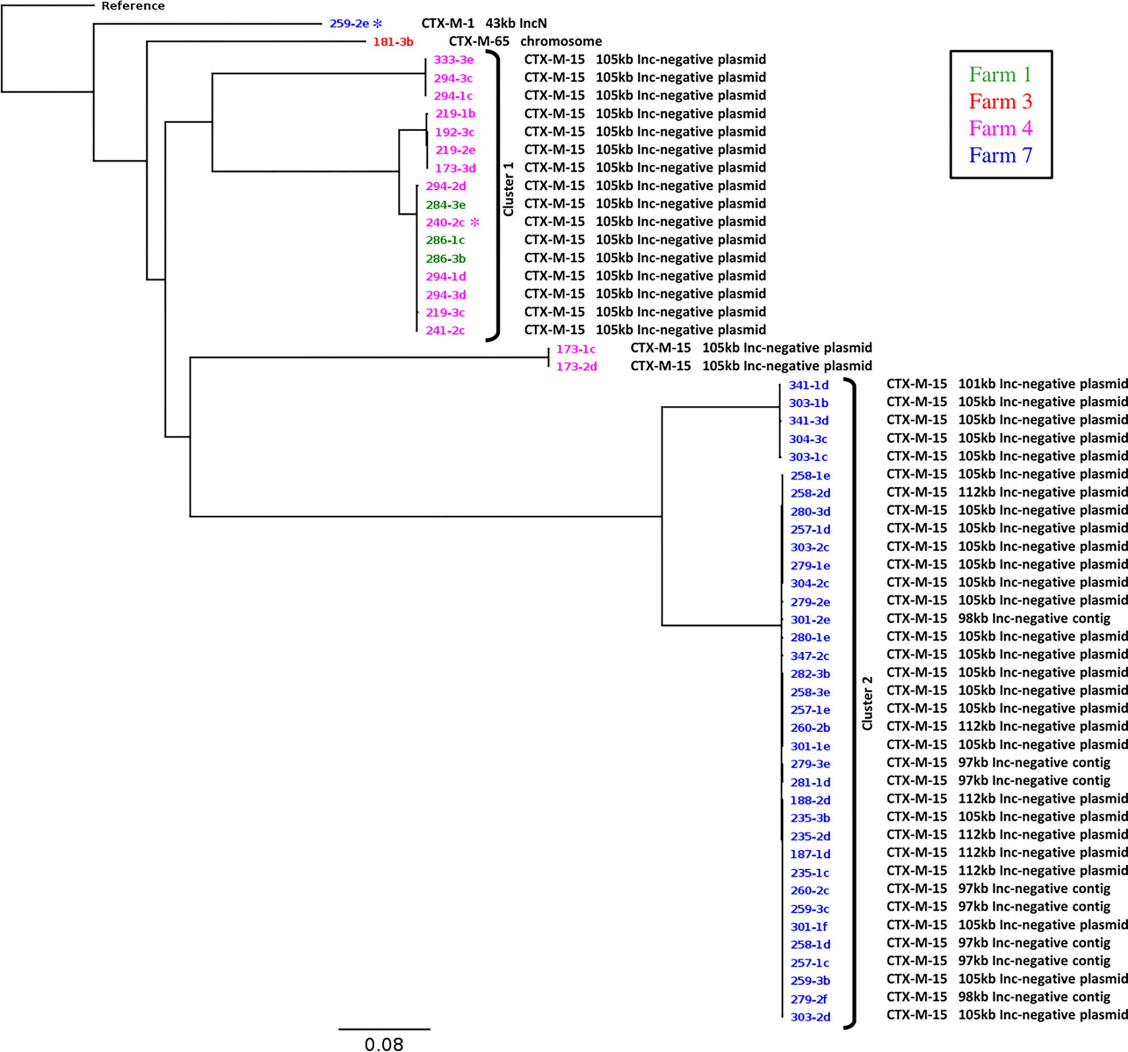
Core SNP phylogenetic tree of all CTX-M carrying *Proteus mirabilis* (chromosomal sequences only) generated by Snippy and FastTree, compared with the HI4320 *P*. *mirabilis* reference strain. Isolates with asterix (*) were used as donors in the conjugation experiments. CTX-M variants and their associated plasmid types (where applicable) are listed beside each isolate.

An average of 67X coverage was obtained with long reads (52X minimum) and 40X with short reads (32X minimum). Chromosomal sequences were fully circularized for 50/56 genomes with an average size of 3.86 Mbp. The contigs from the remaining six not fully circularized sequences ranged between 3.32 and 3.84 Mbp. The chromosomal core SNP analysis showed 22,993 SNPs within the 56-isolate dataset vs. the reference genome. The putative relationships of the isolates from this study derived from the core SNPs are presented in Fig 1. Two major clusters emerged (marked with brackets in Fig 1), with the largest (*n* = 36) comprised of isolates only from farm 7, obtained from all sample types and sampling dates (S1 Table). The second large cluster (*n* = 16) contained a subcluster of tightly related isolates from samples of both farms 1 and 4 obtained between December 2018 and August 2019.

**Fig 2:**
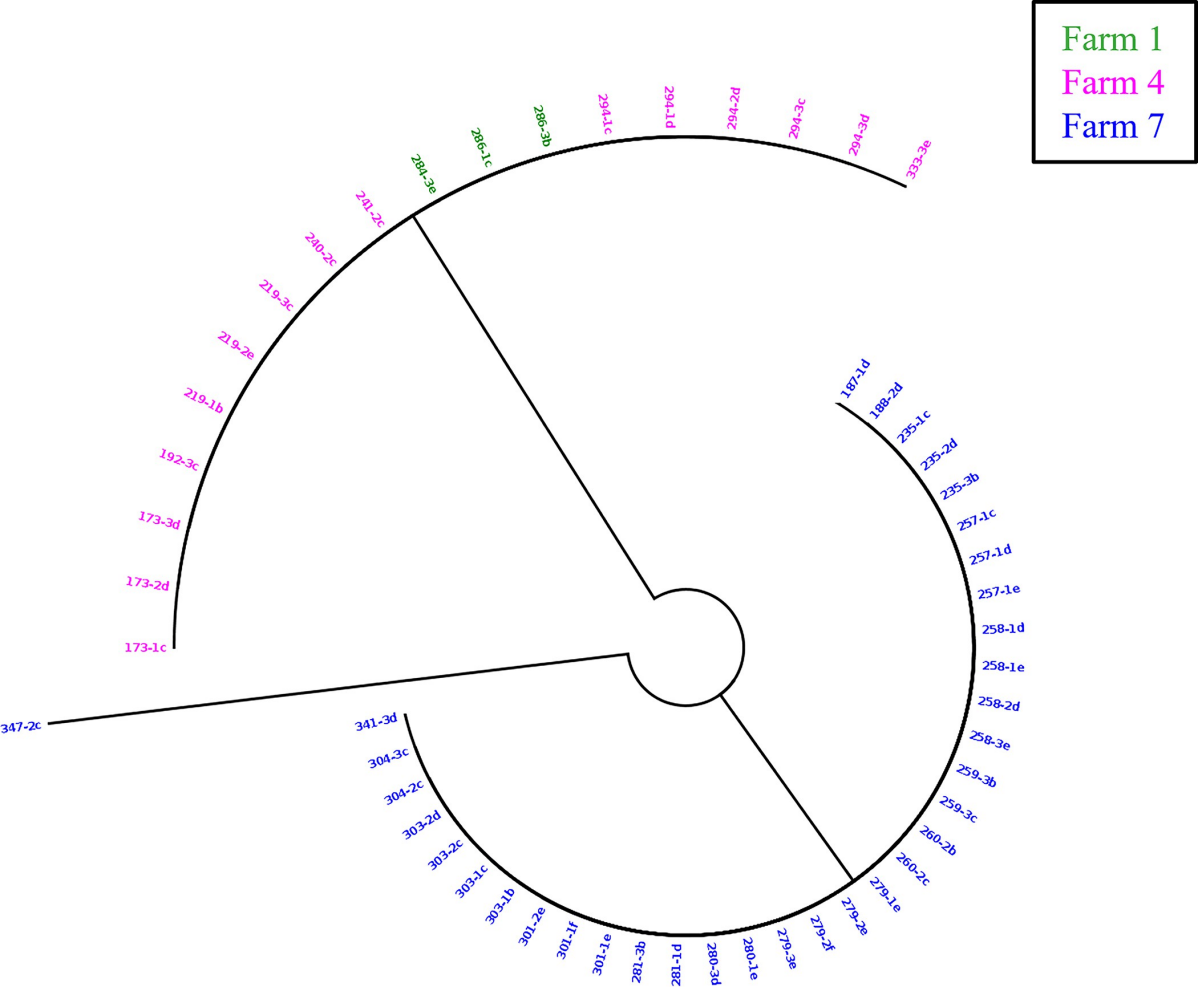
Tree based on core SNP analysis of 53 CTX-M-carrying Inc-negative MOB_H_ plasmids using Roary and SNP-sites.

The majority of the *bla*_CTX-M_-carrying plasmids (54/55) were ~100 kb Inc non-typeable plasmids. The remaining one was a 43 kb IncN plasmid (Table 1). MOB_typer assigned MOB_H_ types to all Inc non-typeable plasmids, and MOB_F_ to the single IncN plasmid. Eight of the plasmids were not completely circularized with Unicycler; contigs between 97kb and 98kb were found but could not be closed due to insertion elements and repeated region interference. The Roary analysis for the 53 comparable Inc-negative plasmids showed 101 shared core genes, 29 shell genes, and 154 total genes considered. A gene presence and absence list is presented in S3 Table. Except for one plasmid (isolate 341-1d) which differed from the others with 59 to 60 SNPs (not included in Fig 2), only 2 SNPs were found among the core gene sequences of the MOB_H_ plasmids; the phenetic tree based on these SNPs is shown in Fig 2.

The 105 kb *bla*_CTX-M-15_/Inc-negative plasmids from the present study were 99.998% identical to pT267A from Tran et al. [10], and the *bla*_CTX-M-1_/IncN plasmid very similar to pT199A (99.920% identity) from the same publication. The complete annotation and structure of these two plasmids can be found in Fig 4C and 4F of the publication [10]. Using BLASTn, only partial sequence coverage with other published plasmid sequences available on GenBank was found. For the *bla*_CTX-M-15_/Inc-negative plasmid, between 62% and 79% coverage (with >99% identity) was found with published plasmid sequences from other *P*. *mirabilis* (e.g. MH491967.2 and CP045540.1). For the 43kb *bla*_CTX-M-1_/IncN plasmid, nearly identical plasmids (>99.8% identity) have been characterized in *E*. *coli* and *Salmonella* isolates from other sources; a *Salmonella* Heidelberg isolate from a 2011 Canadian turkey (CP043224.1) carried this CTX-M-1 plasmid, as well as an *E*. *coli* isolate from a turkey in the USA (MW349106.1).

### Conjugation and stability results

The *P*. *mirabilis* CTX-M-15- and CTX-M-1-carrying plasmids were successfully transferred by conjugation into the *E*. *coli* recipient strain used by Tran and collaborators [10], in both solid media and broth assays. Conjugation frequency on solid media (transconjugant to recipient ratio) was very high (1:2.1 at 30°C and 1:5.3 at 37°C) for the *bla*_CTX-M-15_/non-Inc typeable plasmid, and slightly lower (1:8.8 at 30°C and 1:16.0 at 37°C) for the *bla*_CTX-M-1_/IncN plasmid. Stated differently, the *bla*_CTX-M-15_ plasmid transfer rate was between two and three times higher compared to the *bla*_CTX-M-1_ plasmid, and the transfer rate increased for both plasmids by approximately three to four-fold at 37°C when compared to 30°C. Conjugation efficiency in broth culture was lower (1:12.7 at 30°C and 1:13.9 at 37°C) for the *bla*_CTX-M-15_/non-Inc typeable plasmid, and 1:11.6 at 30°C and 1:10.8 at 37°C for the *bla*_CTX-M-1_/IncN plasmid. Temperature did not seem to have an effect on plasmid transfer efficiency in broth. Transferred plasmids appeared to be stable in the *E*. *coli* transconjugant strains, with all observed colonies tested able to grow on ceftriaxone-containing media after 6 rounds of subculture.

## Discussion

The epidemiology of ESC-resistant *P*. *mirabilis* in animals in Canada and their role as a reservoir of ESC-resistance plasmids has not been well characterized. However, the transmission of *P*. *mirabilis* between animals and humans by consumption of contaminated food or by close contact with animals or their food products has been previously described [27,28]. The primary objective of this study was therefore to characterize ESC-resistant *Proteus* isolates from dairy cattle manure, a potential source of contamination for the human environment and food, and to characterize their *bla*_CTX-M_-carrying plasmids. This provides a comparison basis with similar plasmids from other Enterobacterales also found in manure and in other sources.

ESC-resistant *Proteus* could not be recovered from dairy manure in all six farms investigated. This suggests that ESC-resistant *Proteus* may be less frequent and therefore less worrisome than ESC-resistant *E*. *coli* which were recovered in all of them and in a much larger proportion of samples [11]. The majority of ESC-resistant isolates carried a *bla*_CTX-M_ gene, while only a smaller number of isolates carried a *bla*_CMY_ gene. This contrasts with *E*. *coli* which carried *bla*_CMY_ more frequently than *bla*_CTX-M_ in these same farms [11]. This suggests a different epidemiology for each ESC resistance determinant, depending on bacterial species, even when residing in the same farm and manure environment.

Genome sequencing of CTX-M-positive isolates demonstrated that, similarly to *E*. *coli* [11], some *P*. *mirabilis* clones can colonize an entire manure processing facility and persist in it over extended periods of time. More surprising, was the finding that highly similar *P*. *mirabilis* isolates (zero to three chromosomal core gene SNPs) could be found in two different farms. Thus, unexplained epidemiological links may be present between manure treatment systems of *a priori* unrelated farms that may be worth investigating further.

The low diversity of CTX-M variants with an overwhelming majority of *bla*_CTX-M-15_ observed across the different unrelated *P*. *mirabilis* lineages identified suggests frequent transfer of plasmids carrying this gene. This is in agreement with the extremely low diversity evidenced among the plasmids carrying this gene sequenced in the frame of this study. The only known plasmid closely related to the dominant 105 kb MOB_H_ Inc non-typeable *bla*_CTX-M-15_ plasmid present in the majority of *P*. *mirabilis* isolates in this study has been found by Tran and collaborators when investigating the same farms and samples [10]. However, since it was recovered only after conjugation experiments with a laboratory *E*. *coli* strain, the exact bacterial source of this plasmid in the study of Tran and collaborators remained unknown. The results of the present study strongly suggest that this source could have been *P*. *mirabilis*. This hypothesis is further supported by the very high transfer rate of this plasmid that we obtained between a representative *P*. *mirabilis* isolate from the present study and the *E*. *coli* recipient strain used by Tran and collaborators [10]. Finally, partial homology was observed between the main *bla*_CTX-M-15_-carrying MOB_H_ plasmids found here and plasmids from bacteria isolated in China, including a *P*. *mirabilis* plasmid [29]. Since they have apparently not been detected regularly in more frequently investigated bacterial species, including *E*. *coli* from the same samples as the present study [11], the MOB_H_ plasmids found here may be specific to *Proteus*. Metagenomic studies are clearly needed to explore these hypotheses further and to understand if other bacterial species not studied here represent an even larger reservoir than *P*. *mirabilis* for these MOB_H_ CTX-M-15 plasmids.

In conclusion, the results of the present study show that *P*. *mirabilis* represents an additional bacterial host for the important *bla*_CTX-M-15_ ESC resistance determinant. Although not as frequent as strains of other frequently investigated species carrying this gene, *bla*_CTX-M-15_-positive *P*. *mirabilis* strains can persist in dairy manure treatment systems over extended periods of time. In *P*. *mirabilis*, *bla*_CTX-M-15_ may be present on a type of plasmids not yet widely established in more commonly studied bacterial species. Further studies are needed to investigate whether, despite the high transferability of this plasmid, *P*. *mirabilis* is its main host or it is also established in other less frequently investigated bacterial species.

## Supporting information

S1 Table Characteristics and source information for Proteus mirabilis isolates carrying blaCTX-M (n = 56) from this study. (XLSX)

S2 Table Characteristics and source information for Proteus mirabilis isolates carrying blaCMY (n = 16) from this study. (XLSX)

S3 Table Roary analysis for 53 comparable Inc-negative plasmids showing 101 shared core genes, 29 shell genes, and 154 total genes considered. (XLSX)
